# The effect of edaphic factors on the distribution and abundance of ants (Hymenoptera: Formicidae) in Iran

**DOI:** 10.3897/BDJ.9.e54843

**Published:** 2021-01-15

**Authors:** Mohammad Reza Mohseni, Shahrokh Pashaei Rad

**Affiliations:** 1 Department of Animal Science, Faculty of Basic Sciences, Science and Research Branch, Islamic Azad University, Tehran, Iran (mrmohseni1992@gmail.com), Postal address: Science and Research Branch, Islamic Azad University, Daneshgah Blvd, Simon Bulivar Blvd, Tehran, Iran, Post Code: 1477893855 ORCID ID: 0000-0003-2996-2601, Tehran, Iran Department of Animal Science, Faculty of Basic Sciences, Science and Research Branch, Islamic Azad University, Tehran, Iran (mrmohseni1992@gmail.com), Postal address: Science and Research Branch, Islamic Azad University, Daneshgah Blvd, Simon Bulivar Blvd, Tehran, Iran, Post Code: 1477893855 ORCID ID: 0000-0003-2996-2601 Tehran Iran; 2 Department of Animal Science and Marine biology, Faculty of Life Science & Biotechnology, Shahid Beheshti University, Tehran, Iran (sp2191@gmail.com), Postal address: Shahid Beheshti University, Velenjak, Tehran, Iran, Postal code: 1983969411 ORCID ID: 0000-0001-9387-3166, Tehran, Iran Department of Animal Science and Marine biology, Faculty of Life Science & Biotechnology, Shahid Beheshti University, Tehran, Iran (sp2191@gmail.com), Postal address: Shahid Beheshti University, Velenjak, Tehran, Iran, Postal code: 1983969411 ORCID ID: 0000-0001-9387-3166 Tehran Iran

**Keywords:** biodiversity, Hymenoptera, ecology, edaphic factors, vegetation, Iran

## Abstract

The current study is aimed at investigating the effect of edaphic factors on the distribution and abundance of ants in different habitats of the central areas of Iran, while considering the vegetation. During 2018 to 2019, 20 stations from four habitats, including deserts, mountainous and submontane, plains and rural areas and urban areas, were selected. In general, a total of 311 sample units were collected from all the stations, out of which, 32 species belonging to 13 genera, nine tribes and three subfamilies were identified. The biological distribution and abundance of species were argued by computing the physical and chemical parameters of the soil, such as salinity, pH, total nitrogen, organic carbon, calcium and vegetation. The present study has demonstrated that the calcium content significantly affects the species richness of ants, although the impact of this element on various genera is different. We found that increasing in the abundance and richness of plant species has a positive impact on the abundance and richness of ants. Our results also show that some genera are meaningfully adaptable to a variety of habitats. In Kahak station, which is an urban habitat, with enormous diversity, 14 species were found, while in Sadrabad Historic Karvansara, a desert habitat, only *Cataglyphis lividus* (André, 1881) was collected. *Cataglyphis bellicosus* (Karavaiev, 1924), as the most abundant species, collected from 12 stations, was the most dominant species.

## Introduction

Ants (Hymenoptera: Formicidae), being high in the abundance of species in nature, play a determining role in ecosystems and their biodiversity. Since ants can feed on plants and plant products, such as sap or are predators, ant biodiversity is often of considerable importance (Agosti and Johnson 2003, Sanders and van Veen 2011).

Ants act as ecosystem engineers and can change the physical and chemical factors of the soil (Jilkova et al. 2010). These changes, in turn, play an essential role in the development of living organisms (Folgarait 1998, Dostál et al. 2005).

The habitat heterogeneity hypothesis is regarded as one of the current hypotheses to explain variations in species diversity at the local scale, by stating that structurally complex habitats offer better support for more species as they arrange for more niches and means of exploiting the available resources (Tews et al. 2004). This hypothesis can be often used to explain diversity patterns at the landscape scale, where landscapes with a diversity of habitat kinds should support more species than those comprising a single homogeneous habitat (BÖhning-Gaese 1997; Tews et al. 2004). Most studies of ant diversity have assessed the ecological factors that affect species richness within habitats (Kaspari 1996; Sarty et al. 2006; Parr 2008; McGlynn et al. 2009) or across all regions (Kaspari and Weiser 1999; Dunn et al. 2009; Vasconcelos et al. 2010). However, many aspects of this subject, notably the impacts of soil parameters and vegetation, are still unclear and need further investigations.

Edaphic factors and vegetation changes are predicted to influence the distribution and abundance of ants in many ways. They could affect communities both directly, by means of changes in behaviour and physiology and indirectly, such as variations the host plants go through in their biochemistry (e.g. Yuan et al. 2009), morphology (Barnes et al. 1988, Lake and Wade 2009), physiology (Gifford et al. 1996, Yadugiri 2010) and patterns of abundance, diversity and richness (Thuiller et al. 2005, Kazakis et al. 2007).

The limits of higher and lower salinity tolerance restrict an ant’s performance (reviewed by Andersen 1995), for instance, limiting its abundance, survival and distribution (Gray et al. 1997). Therefore, differences in abundance and distribution patterns rest partially on the diverse ecological factors of ant species (Pol and de Casenave 2004).

It has been proven that the attributes of both the edaphic factors and vegetation cover affect distribution and abundance of ants. Some studies (Terayama 1992, Quiroz-Robledo and Valenzuela-Gonzalez 1995, Torres and Snelling 1997) found that the best predictors of ant species richness are often abundance and richness of the plant community. Some studies have shown an inverse correlation between plant biomass and ant diversity (Caldas and Moutinho 1993, Parr et al. 2002), while others have suggested a positive correlation between invertebrate diversity and that of the plant (Dean and Milton 1995, Picker and Samways 1996). However, for other insect groups, such as butterflies, no correlation has been observed between plant variables and species richness and abundance (Hawkins and Porter 2003). Nevertheless, only a few of these studies investigated the edaphic factors, a third variable affecting both plant and ant communities. Many studies point out at least one of numerous plant attributes (e.g. percentage of cover, biomass richness) as a causal mechanism which influences ant diversity (Caldas and Moutinho 1993, Dean and Milton 1995, Picker and Samways 1996). Still, the edaphic factors may be equally, if not more significant, than plant attributes in explaining ant diversity patterns, since most ant species nest in the soil (Boulton et al. 2005).

The ecological significance of ants and their diversity (Folgarait 1998) motivated community researchers to investigate patterns of species composition and ant distribution all over the world. Species richness and ant composition vary between habitats (Andersen 1995) with distinctive ecological circumstances. Nevertheless, there is not much information about the factors that have an impact on the composition of ant assemblages. Ants which are found in Central Asia, in particular, have been overlooked, with few studies taking into account Iran’s ant fauna and factors that control its composition.

Research on ant assemblages in different habitats of Iran is necessary not only to complete the ants' fauna of this area, but also to understand population dynamics of this group of insects. A few studies addressing abundance and diversity of ants in Iran are available (e.g. Paknia et al. 2008; Mohseni 2018; Mohseni and Pashaei Rad 2019).

Since Iranian scholars have mainly presented their taxonomical and ecological reports at specific national scientific congresses or published them in local journals, it is hard for foreign biologists to have access to this literature. Other problems are caused by old records which require revision. This research can be a helpful reference for myrmecologists and ecologists fascinated by Asian ants and comprehending the influence of edaphic factors on ant communities.

In this study, we explored the variation in species richness, abundance and species composition of ants across different habitats including desert, mountain and submontane areas, plains and human settlements in central Iran. We also investigated edaphic factors by considering vegetation that potentially influence ant communities. This study's other primary purpose was to complete the knowledge of ants and the ant fauna of Iran.

These results are essential to promoting better ecological management procedures and preservation. Regarding growing environmental problems due to habitat loss, environmental monitoring techniques were developed through biological indicators (Hunter 2002; Henry et al. 2007). For example, as significant and dominant plain members, ants can be used as indicator species for monitoring the management practices and conservation (Underwood and Fisher 2006; Moranz et al. 2013). Not much information is available for considering the role of ants in temperate plains, their main role in soil nutrient cycling, microbial community, plant community regulation and pest suppression (Frouz et al. 2003; Nemec 2014). The present study results are fundamental for later long-term monitoring plans and could aid protocols for early warnings of global environmental change influences on biodiversity.

## Material and methods

### Study area

This study concentrated on the central areas of Iran, nearly 2800 km^2^, with various environmental conditions made available by a range of vegetation formations and climatic belts. This area has a latitudinal range that spreads into the plains, mountainous and rural areas, salt pans, foothills, urban habitats and deserts. A longitudinal range creates changes in woodland composition because of a falling gradient in rainfall from the Caspian Sea in the north to the interior and an altitudinal range between extensive mountain chains of the Zagros and Alborz in the west and north, respectively.

**Figure 1.**
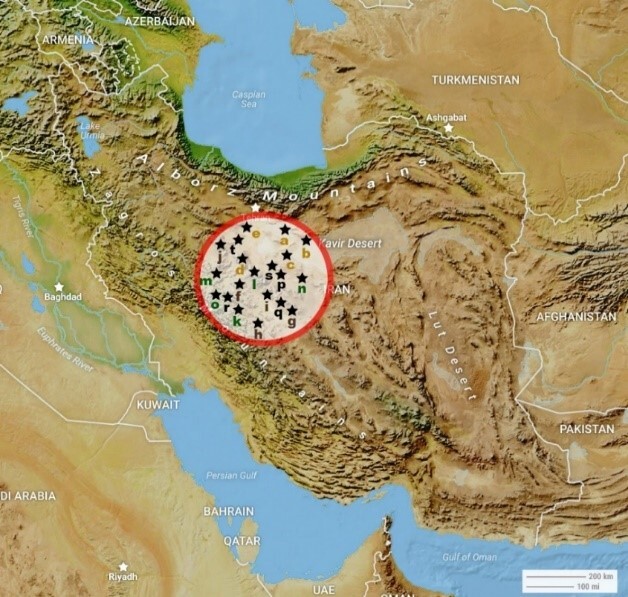
The location of sites in the centre of Iran (sites are named, based on Suppl. material 1). a = Salt Lake, b = Historic caravanserai of Sadrabad, c = Salty-lands of Qom Rood, d = Tagharood industrial area, e = Cheshmeh Palang Village, f = Darbandshoor Mount, g = Shah Ismaeil shrine, h = Chalk mine, i = Kebar Dam, j = Ghahan Village, k = Cheshme Ali Village, l = Ghadir Forest Park, m = Varzaneh Village, n = Qanavat City, o = Dastjerd City, p = Qom City, q = Kahak City, r = Salafchegan City, s = Kamkar Castle, t = Jafariyeh City

**Figure 2.**
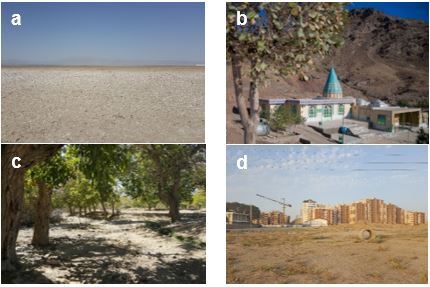
Habitats under study a) Desert; b) Mountainous and submontane; c) Plain and rural; d) Urban

Different areas in central Iran with altitudes and latitudes of 0.80 ± 50.90 and 0.36 ± 34.60 (Fig. 1) were divided into 20 stations from four habitats including deserts with hot and dry climate and sparse vegetation; mountainous and submontane areas with warm and semi-mild climate and sparse vegetation; plains and rural areas with semi-mild and semi-hot climate with vegetation and the urban habitat with semi-warm and semi-arid climate with relatively sparse vegetation (Fig. 2).

We worked on five sampling sites with different ecological conditions for each habitat. All of the five locations were selected, based on their unique conditions to find a maximum variety of ant species. All sites-related factors are shown in Table 1.

### Sampling methods

Sampling was carried out in 20 sites (Table 1) during spring, summer and autumn, 2018 and 2019, using pitfall trapping and by other sampling protocols, such as hand collecting and sweep netting (used in sites with rich vegetation) at fixed time periods.

The samples of each site were taken twice in each season through pitfalls, hand collecting and sweep netting (in some cases). To investigate and study ant abundance, sampling was conducted once in the morning (8 am to 12 noon) and the second in the afternoon (5 p.m. to 8 p.m.).

Sampling was carried out by traps made of transparent cups that contained sugar syrup and propylene glycol (Sierra antifreeze). Pitfall traps (9 oz Solo® plastic cups, 7 cm in diameter) were randomly located at a distance of 25 m from each other in a regular grid plot of 25 m x 25 m. It was carried out to guarantee the samples' independence and lessen individuals' probability from another plot falling into the trap of the target plot. Pitfall traps were set out for 48-hour periods. The numbers of pitfall traps used varied from year to year. Five pitfall traps per site in 2018 and four pitfall traps per site in 2019 were designed and implemented. In this study, two trapping patterns were used: (i) Typical simple pitfall and (ii) X-shaped guidance barrier pitfall using four 50 cm long wooden barriers (height: 8 cm angle: 90).

Sweep netting was also utilised to collect ants on herbs and shrubs. Heavy-duty muslin nets were also used to standardise one complete sweep as a figure-eight movement of the net through vegetation. As with the pitfall traps, the sampling intensity by sweep netting varied from year to year. Three times of sweep netting at each site in 2018 and two times of sweep netting at each site in 2019 were designed. Sweep netting was used at the beginning and end of the day.

In this method, transects stretching in sites with reasonable vegetation cover were demarcated and divided into ten (6 m × 5 m) sections. Ant density was collected in three or two (variable according to the vegetation cover of the sites) randomly selected 3 m^2^ quadrats per section by sweep netting for 2 minutes.

There were limitations in the sampling process because of exceptional climatic conditions in central parts of Iran, such as extremely hot days during dry months (June, July, August and September) and heavy rains in the wettest month (October). The recorded temperature ranges of sites from the sampling times are shown in Table 1.

### Identification of Samples

The samples were collected in tubes containing 80% ethanol, transferred to the biosystematics laboratory of Shahid Beheshti University of Tehran and then identified to subfamily, genus and species with the NSZ-405 stereomicroscope and identification keys of Bolton (1994), Collingwood (1985), Goulet and Hubert (1993), Radchenko (1998) and Hashimoto (2003). All the identified species were reviewed and verified by Dr. Brian Taylor, Royal Entomological Society of London, United Kingdom. The voucher specimens of the species have been archived with the Shahid Beheshti University of Tehran, Iran.

### Environmental variables

#### Vegetation types in each site

The plant specimens with all their parts of sites were collected in an attempt to identify and report the flora present in the sampling sites. Dr. Mehrabian, Department of Botany at Shahid Beheshti University, Tehran, identified the plant specimens collected at each sampling site (Table 2). All collected plant specimens were archived in the Shahid Beheshti University of Tehran, Iran.

For collecting smaller specimens, the quadrat sampling (plots of a standard size) method was utilised. Significant aspects of plant community measured by quadrat sampling are included into frequency, density and cover (Cox 1990). A quadrat sets the limits in an area where vegetation cover can be plants counted, estimated or species listed. The collecting area size is regarded large enough to encompass considerable numbers of individuals, but not trivial. The plants can be separated, counted and measured without omission or duplication of individuals. Since plant numbers in each unit area had to be measured, then the quadrat size was vital. Quadrats 50 cm x 50 cm (type 1) for long grass or heathland and other low-growing vegetation, 25 cm x 25 cm quadrats (type 2) for short grassland and 10 cm x 10 cm quadrats for tiny plants were considered. Considering the sorts of vegetation cover per site, the quantity of the type of quadrats used varied from one to two. For larger specimens, trees and shrubs, plots on the ground were set out using tape measures.

#### Physical and chemical parameters of soil in sites

At each site and year, soil A-horizon depth (mm), soil compaction (Lang Penetrometer Units) and soil shear stress (kg/cm^2^) were sampled to identify the texture and provide an approximation of certain soil elements at the sites. Soil samples were collected from areas adjacent to the colonies and the closest spots to the ant collection points. Next, the samples were transferred to plastic bags in the field and were taken to the laboratory for chemical analysis.

Various tests on the chemical and physical parameters of soil, such as texture (% of silt, sand and clay), salinity, electrical conductivity (EC), pH and organic carbon, total nitrogen content, magnesium, calcium, absorbable phosphorus, sodium, absorbable potassium and sodium absorption ratio were conducted at all the sampling sites (Suppl. material 2).

By using an EC meter to measure salinity, a pH meter to measure alkalinity and a flame photometer to measure sodium and potassium, physical and chemical parameters of the soil at sampling sites were investigated and a spectrophotometer was utilised to determine the amount of phosphorus. A titration was also used to check the amount of calcium and magnesium. A titration with the colour variation of the Ortho-Phenanthroline ferrous technique was applied for the measurement of organic carbon. We used a hydrometer for the detection and analysis of the soil texture.

The collection, testing and analysis of physical and chemical soil parameters were conducted to evaluate the potential effect and correlation of measured physical and chemical factors on the population, diversity and abundance of the ant species in their habitats.

### Data analyses

Ant species richness or diversity over locations was calculated using diversity indices including species richness (S), Shannon–Wiener diversity index (H´) and Pielou’s evenness (J´) (Jorgensen et al. 2005) in PRIMER (v6) software (Plymouth Routines in Multivariate Ecological Research, Plymouth Marine Laboratory, Plymouth, UK; Clarke and Gorley 2006). The hypothesis that taxonomic richness, abundance, Shannon–Wiener diversity index (H´) and Pielou’s evenness (J´) for ants was significantly different amongst habitat types was tested by one-way univariate analysis of variance (ANOVA) with one factor including habitat type in four levels (i.e. desert, mountainous and submontane, plains and rural and urban). Pairwise comparison between habitats was tested by the SNK test. The Shapiro-Wilk test was used to test if data were normally distributed and results showed that data on species richness and Shannon–Wiener diversity were normally distributed, but data on abundance and Pielou’s evenness were not normally distributed. The normality of data abundance was achieved by a square root transformation. Since none of the transformation methods achieved normality for Pielou’s evenness measure, a significant difference in Pielou’s evenness measure amongst habitat types was tested by the Kruskal-Wallis test, followed by the Mann-Whitney U test for pairwise comparisons, using SPSS software, v. 26.

Non-metric Multidimensional Scaling (nMDS) constructed on the Jaccard resemblance measure was used to delineate the pattern of species composition across habitats (Clarke and Warwick 2001). Likewise, nMDS constructed on Bray–Curtis similarity matrix was used to delineate the assemblage pattern. The significance of differences in ants assemblage structure amongst habitat types was examined by one-way permutational multivariate analysis of variance (PERMANOVA). In the case of significant differences in communities across habitats, a similarity percentage analysis (SIMPER) was used to identify the species or group of species which contributed most to the dissimilarities amongst habitats (Clarke and Warwick 2001). The multivariate analyses were conducted using PRIMER (v.6) software (Plymouth Routines in Multivariate Ecological Research, Plymouth Marine Laboratory, Plymouth, UK) (Clarke and Gorley 2006).

A Canonical Correspondence Analysis (CCA) in Canoco5 (Ter Braak 2002) was used to identify the subset of environmental variables that were potentially structuring ant assemblages across sites within habitats. The response data (ants) were compositional and had a gradient of 4.1 SD units long, so the unimodal method (CCA) was suggested by Canoco5. The CCA was applied to the dataset of 12 explanatory variables (i.e. Na, Ca, Mg, K (ava), P (ava), O. C., total N, pH, salinity (EC) and the percentage of sand, silt and clay in the soil and four supplementary variables, including the four habitat types (i.e. desert, mountainous and submontane, plains and rural and urban). A manual forward selection process in Canoco5 was used to select the subset of environmental variables. Prior to analysis, environmental variables and species density data were logged and the square root transformed, respectively.

## Results

During the spring, summer and autumn in 2018 and 2019, a total of 311 sample units were collected from the 20 sites and contained 32 species that belonged to 13 genera, nine tribes and three subfamilies of Myrmicinae, Formicinae and Dolichoderinae.

The species of *Cataglyphis bellicosus* (Formicinae) was collected from 12 sites (37 sample units) in the central regions of Iran; therefore, it was indicated as the dominant species of these areas. *Tapinoma simrothi* (Krausse, 1911) was collected, as the only species of Dolichoderinae, from eight sites. *Messor galla* (Mayr, 1904), *M. rufotestaceus* (Foerster, 1850), *M.* sp. (Forel, 1890), *Monomorium pharaonis* (Linnaeus, 1758), *Tetramorium moravicum* (Kratochvíl, 1941), *Crematogaster oasium* (Santschi, 1911) (Myrmicinae) and *Cataglyphis altisquamis* (André, 1881), *C. frigidus* (André, 1881), *Lepisiota bipartita* (Smith, F., 1861), *Plagiolepis abyssinica* (Forel, 1894), *Cardiocondyla stambuloffi* (Forel, 1892), *Paratrechina longicornis* (Latreille, 1802) and *Camponotus flavomarginatus* (Mayr, 1862) (Formicinae) had the lowest abundance, each collected at only one site (Suppl. material 1).

**Figure 3.**
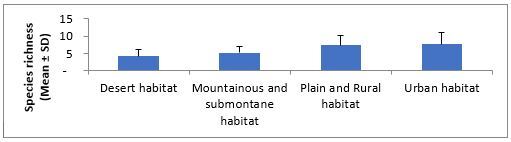
Variation in ant’s species richness across different habitat types

**Figure 4.**
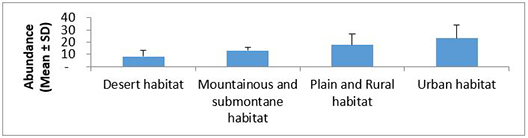
Variation in ant’s abundance across different habitat types.

**Figure 5.**
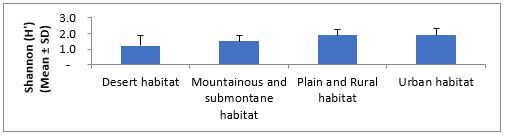
Variation in ant’s Shannon (H') measure across different habitat types.

**Figure 6.**
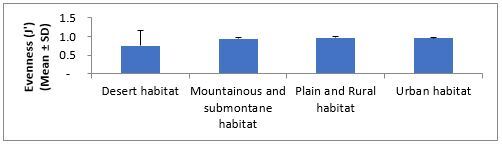
Variation in ant’s evenness (J') measure across different habitat types.

The order of average species richness, abundance, Shannon–Wiener and evenness measures ranged from the lowest to highest values in desert, mountainous and submontane, plains and rural and urban habitats (Fig. 3, Fig. 4, Fig. 5 and Fig. 6), respectively. The mean abundance measured in our study was calculated by averaging the abundance (i.e. number of species occurrences) of all species of all replicate samples in each habitat type. The results of one-way ANOVA showed no significant differences in average species richness (F = 2.03, df = 3, P = 0.15) and Shannon–Wiener Index (F = 2.20, df = 3, P = 0.13) amongst habitat types. The average abundance of ants was significantly different amongst habitat types (F = 4.02, df = 3, P = 0.03). Further pairwise comparisons showed that the average of ant abundance in the urban habitat was significantly higher than that of desert habitat, but the average abundance of ants in mountainous and submontane habitat and plains and rural habitat showed no significant differences from those in urban and desert habitats. No significant difference was found in Pielou’s evenness measure amongst habitat types (Kruskal-Wallis H = 6.21, df = 3, P = 0.10).

**Figure 7.**
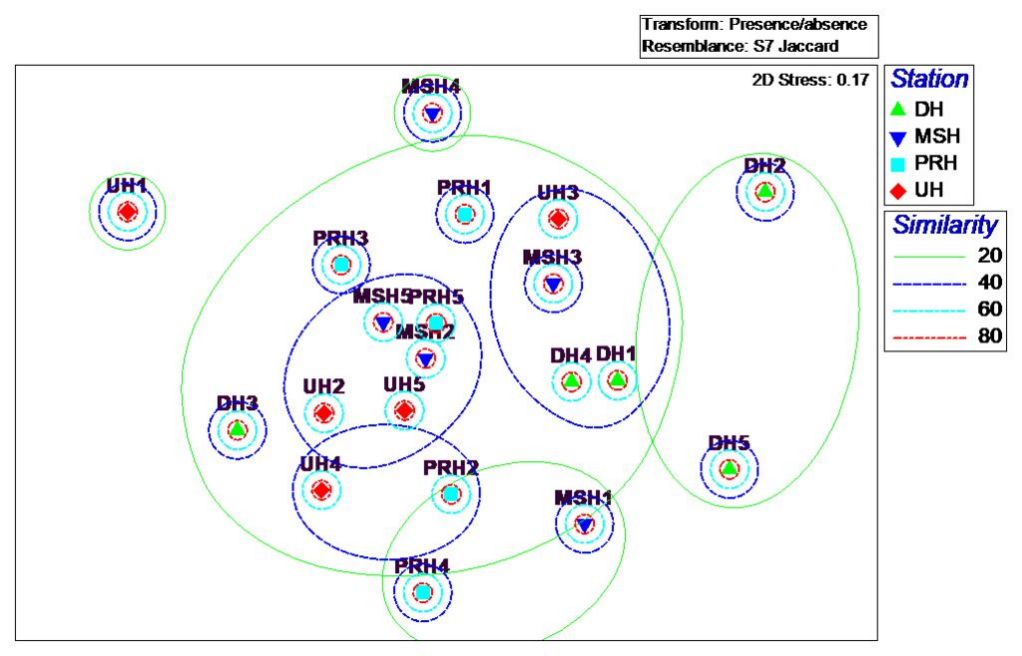
nMDS plot of species composition, based on Jaccard distance measure derived from presence/absence of ant’s species in four habitats. DH = desert, MSH = mountainous and submontane, PRH = plain and rural, UH =urban. DH1 = Salt Lake, DH2 = Historic caravanserai of Sadrabad, DH3 = Salty-lands of Qom Rood, DH4 = Tagharood industrial area, DH5 = Cheshmeh Palang Village, MSH1 = Darbandshoor Mount, MSH2 = Shah Ismaeil shrine, MSH3 = Chalk mine, MSH4 = Kebar Dam, MSH5 = Ghahan Village, PRH1 = Cheshme Ali Village, PRH2 = Ghadir Forest Park, PRH3 = Varzaneh Village, PRH4 = Qanavat City, PRH5 = Dastjerd City, UH1 = Qom City, UH2 = Kahak City, UH3 = Salafchegan City, UH4 = Kamkar Castle, UH5 = Jafariyeh City.

**Figure 8.**
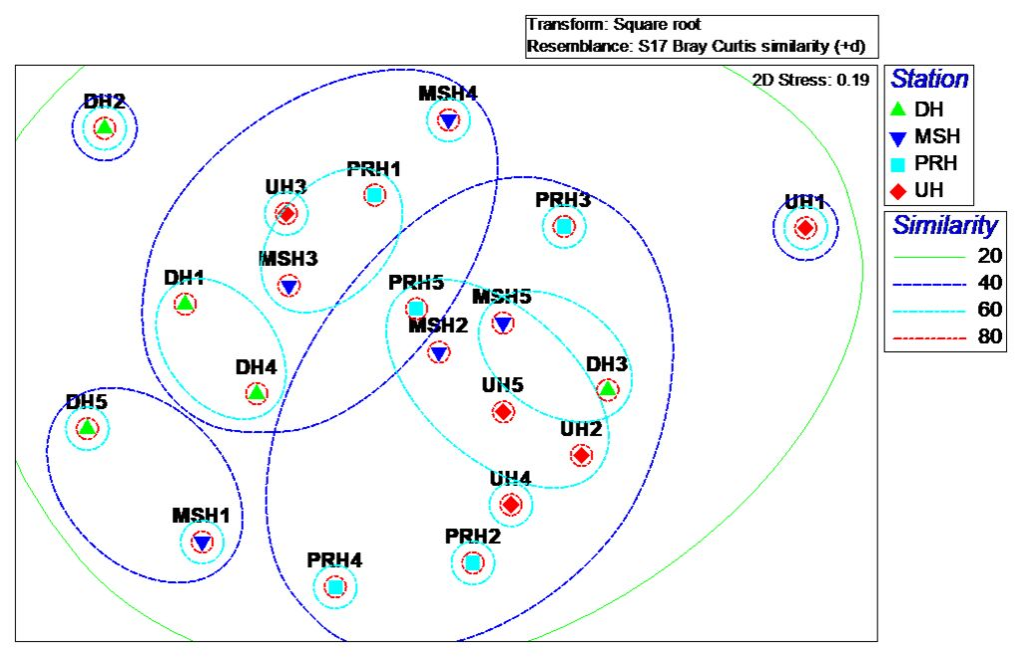
nMDS of ant’s assemblage structure based on Bray–Curtis index of similarity derived from the square root transformed means of abundance data in four habitats. DH = desert, MSH = mountainous and submontane, PRH = plain and rural, UH = urban. DH1 = Salt Lake, DH2 = Historic caravanserai of Sadrabad, DH3 = Salty-lands of Qom Rood, DH4 = Tagharood industrial area, DH5 = Cheshmeh Palang Village, MSH1 = Darbandshoor Mount, MSH2 = Shah Ismaeil shrine, MSH3 = Chalk mine, MSH4 = Kebar Dam, MSH5 = Ghahan Village, PRH1 = Cheshme Ali Village, PRH2 = Ghadir Forest Park, PRH3 = Varzaneh Village, PRH4 = Qanavat City, PRH5 = Dastjerd City, UH1 = Qom City, UH2 = Kahak City, UH3 = Salafchegan City, UH4 = Kamkar Castle, UH5 = Jafariyeh City.

The nMDS ordination plots of locations (habitats), generated by presence/absence and square root data of abundance, are illustrated in Fig. 7 and Fig. 8. The ordination of nMDS, based on species composition of ants, showed that sites within habitats could be placed in four groups (80% dissimilarity), including (1) one site in urban habitat (UH1), (2) one site in mountainous and submontane habitat (MSH4), (3) two sites in desert habitat (DH2, DH5) and (4) remaining sites (Fig. 7). The ordination of nMDS, based on the assemblage structure of ants, showed that sites within habitats could be placed in four groups (60% dissimilarity), including (1) one site in desert habitat (DH2), (2) two sites in mountainous and submontane (MSH1) and desert (DH5) habitats, (3) one site in urban habitat (UH1) and (4) remaining sites (Fig. 8).

The result of the PERMANOVA test showed no significant difference in assemblage structure of ants amongst habitats (Pseudo-F = 1.25, df = 3, P (perm) = 0.23).

The results of the SIMPER analysis indicated that dissimilarity between paired habitats ranged from 63.15% to 77.74% (Suppl. material 3). The contribution of the top-ten ant species in assemblage structures of paired habitats is presented in Suppl. material 3. The prominent species were *Cataglyphis bellicosus*, *Messor mediorubra* (Forel, 1905), *Lepisiota dolabellae* (Forel, 1911),and *Tapinoma simrothi* (Suppl. material 3).

### Environmental variables

**Physical and chemical parameters of soil in sites**

The highest salinity, total nitrogen, organic carbon and calcium were found in the soil at the Salt Lake site (central part) and the least amounts at the Darbandshoor site. The highest and lowest pH occurred at Darbandshoor and the Salt Lake site (central area), respectively. The variation in physical and chemical parameters of soil as the measured environmental variables is presented in Suppl. material 2

### Environmental variables underlying ant assemblages

Enter subsection text

#### *Physical and chemical parameters of soil sites*

**Figure 9.**
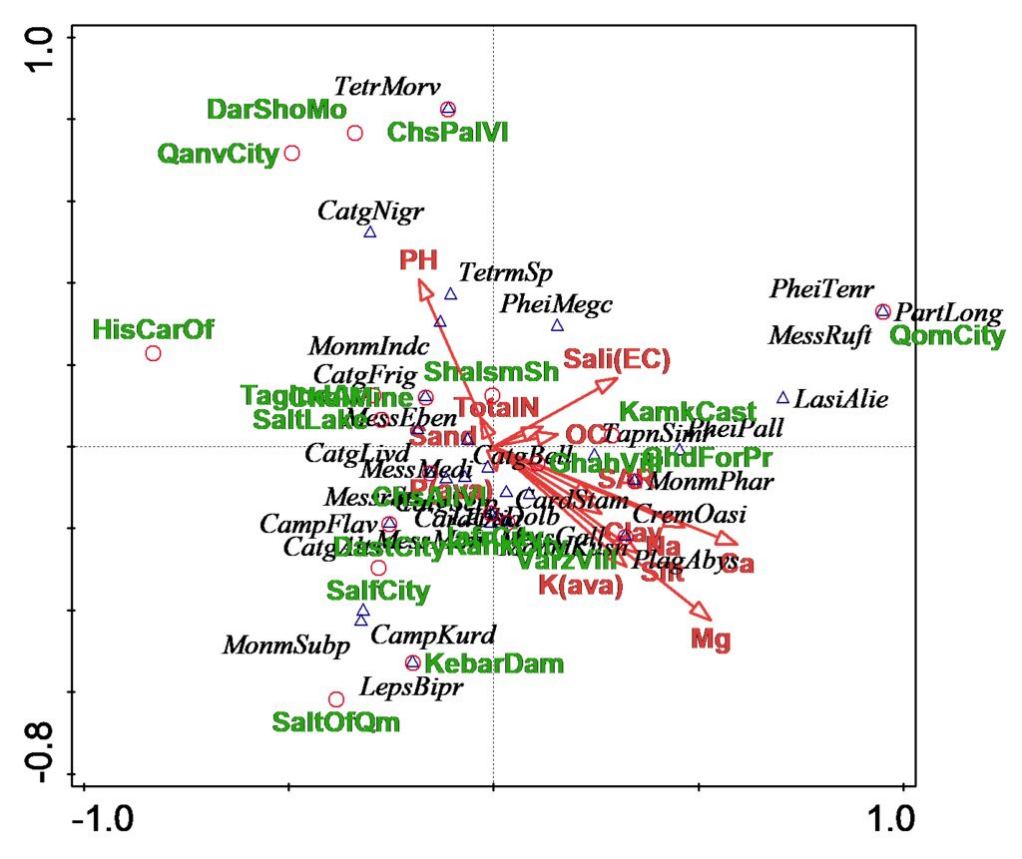
Canonical Correspondence Analysis plot showing an association between each physicochemical variable and sampled ant assemblages, in relation to the first two ordination axes.

The result of CCA demonstrated that only the parameter of soil as the environmental variable (i.e. Ca) was significantly correlated with variation in the spatial distribution pattern of ant assemblages. Using environmental variables as independent variables, axes 1 (λ1 = 0.41) and 2 (λ2 = 0.27) explained 68.5% of the variance in ant assemblages. Based on the manual forward procedure, the calcium content in the soil was significantly (pseudo-F = 1.7, *P* = 0.01) associated with variation in the spatial distribution pattern of ant assemblages (Fig. 9). Calcium was positively correlated with the presence of *Plagiolepis abyssinica* and *Crematogaster oasium* and negatively correlated with the presence of *Monomorium indicum* and *Cataglyphis frigidus* (Fig. 9).

### Vegetation data

In total, 1398 plant specimens were recorded and 134 specimens were gathered, out of which 85 specimens were colonised by the ant colonies. Ant colonies were located at the base, stem tissues and internal duct of branches of these plants. The plant species' height varied from 0.1 m to 3.5 m. All plant species are shown in Table 2.

Plain, rural and mountain habitats with 31 different plant species and desert and urban habitats with 21 and 17 different plant species had the highest and lowest species richness, respectively. Plain and rural habitats with 512 plant specimens, mountain habitat with 313 specimens, urban with 295 specimens and desert habitat with 268 plant specimens had the highest and lowest abundance of plant specimens, respectively.

## Discussion

According to our predictions, the impacts of ecological factors are found in each sampling area.

The results of the present study from four different habitats of the central areas of Iran with entirely different environmental conditions show that, except for calcium, the increasing or decreasing of the other chemical elements of the soils such as salinity, pH, total nitrogen and organic carbon of the soil, does not significantly affect the abundance and richness of ant species.

It seems that in the current study, the presence and interference of very different environmental factors in various habitats, such as very different vegetation coverage and climates, differ entirely from those by Mohseni and Pashaei Rad (2019) studies in salt marshes and salt pans.

Based on the results of Canonical Correspondence Analysis in the present study, indicating the significant effect of calcium on the presence of ant species, we hypothesise that, while there is a significant effect of calcium on the presence of ant species, the impact of this element on various genera is different. This element had a significant positive effect on the presence of *Plagiolepis abyssinica* and *Crematogaster oasium* species and a significant negative effect on the presence of *Monomorium indicum* and *Cataglyphis frigidus* species. Studies conducted by Hill et al. (2008) in north-eastern Mississippi showed that *Solenopsis* spp. from the Myrmicinae subfamily are inclined to nest in more calcium-rich lands than in other areas and reports by Almeida et al. (2019) on the Brazilian Atlantic Forest showed higher concentrations of calcium in the ant nest margins than other regions and the findings of Cammaerts and Cammaerts (2018), in 2018, on the negative effects of calcium compounds on physiological and ethological traits strongly reinforce our hypothesis. Our second hypothesis is that for some genera, which are classified as adapted genera, calcium is considered as limiting macronutrients as well as nest litter and, for some genera, it is regarded as a limiting factor. The findings of Sternberg et al. (2007) on “Macronutrients Accumulated in Leaf-Cutting Ant Nests”, as well as Finér et al. (2013) on “Wood Ants and Nutrient Dynamics” and the study made by Cammaerts and Cammaerts (2018) on adverse effects of calcium compounds on ants’ food consumption, general activity, cognition, trail following, audacity, orientation ability tactile (pain) perception, escaping ability, conditioning, short and middle-term memory, strongly support our second hypothesis while confirming the results of this study.

Reporting on species belonging to *Cataglyphis* (Förster, 1850) from the Kalahari Desert in southern Africa, the Maharès desert areas in Tunisia and the central deserts of Iran with a warm and dry climate, by Heatwole (2001), Wolf et al. (2018) and Mohseni (2018), respectively, confirm the results of the present study and justify the dominance of *Cataglyphis* species, such as *C. bellicosus* and *C. lividus* and species of *Lepisiota* (Santschi, 1926), such as *L. dolabellae*, with longer legs and larger bodies than other species.

Based on the findings of the current study, another dominant species in the central parts of Iran is *Lepisiota dolabellae*, which was collected at 12 different sites with different climates and vegetation covers. The above species was also reported from the northern regions of Iran, with a very humid climate and very dense vegetation, by Paknia et al. (2008) and from Chania, Greece, with a moderate and Mediterranean climate and relatively suitable vegetation cover by Borowiec and Salata (2012). Due to differences in weather conditions in the northern region of Iran and the city of Chania in Greece and the findings of this study, it can be concluded that *Lepisiota dolabellae* species is highly adaptable to different climatic conditions and vegetation covers.

The present study results indicate that the increase in the abundance of plant specimens in natural habitats has a direct positive effect on the abundance and species richness of ants. It is proved by the presence of the highest abundance of specimens and the highest diversity of ant species in the Plain and rural habitat with 512 plant specimens and the lowest number of specimens and richness of ant species in the desert habitat with 268 plant specimens. Studies by Oliveira et al. (2011) and Stephens et al. (2016) in Brazil and Ghana confirm the present study's findings.

However, given the results, we believe that more determining factors, such as the edaphic factors, food resources and environmental conditions for nesting in urban habitats reduce the positive effect of plant species' abundance and richness on the abundance and richness of ants. As the urban habitat has the highest gradient of calcium (Suppl. material 2), the most food sources resulting from co-existence with humans and the most diverse environments for nesting have the largest number of ants in terms of abundance and richness. However, with 295 plant specimens and 17 plant species, the urban habitat is the third habitat with the lowest abundance and the least plant richness compared to other habitats. The studies of Bestelmeyer and Wiens (2001), Wang et al. (2001) and Boulton et al. (2005) regarding the much higher impact of edaphic factors on vegetation and the abundance and richness of ants strongly reinforce our view.

Amongst other findings of the present study is the exceptional symbiotic relationships between ants and plants, which was briefly discussed in the Materials and Methods section. The substantial and dense presence of ants in the vicinity of some plant species and their internal tissues shows the possible particular relationships between ants and plants. Due to the existence of common herbivorous, flea beetle larvae in many sampling areas in the vicinity of these plants, we strongly assume that ant species protect plants against these insects and instead use the internal tissues of plants for nesting and plant nectar for feeding. In some cases, ants of a colony were seen feeding on plant nectar, which could also confirm the symbiosis of ant species with some plants when they use the nectar and protecting trees against pests. Studies conducted by Katayama and Suzuki (2004), Mayer et al. (2014) and Lortzing et al. (2016) strongly confirm the hypotheses of the present study. The second hypothesis is that ants provide services on the molecular scale instead of receiving rewards from plants, such as plant‐produced food and housing. Studies conducted by Offenberg and Damgaard (2019) show that ant-produced antibiotics spread to their host plants and reduce plant pathogenic loads, providing evidence of a special relationship between ants and plants, which firmly confirms our hypothesis. However, other ants' services to plants, such as pollination and soil movement, can also be considered.

Generally speaking, and by taking into consideration similar studies, it can be argued that ants can adapt significantly to different environments with unique conditions. However, some species, such as species of the *Cataglyphis* and *Lepisiota* genus, are unique to specific biological conditions.

Although the authors have tried to evaluate and represent the effects of ecological factors on the distribution and abundance of ants, countless aspects of this issue are still unknown and need further investigations in the future.

## Conclusions

In this paper, results have demonstrated that the biodiversity and species distribution of ants in different habitats with different unique conditions are affected by edaphic factors.

According to the present study, calcium was one of the most influential factors in species distribution. In more detail, this element has a positive effect on the presence of *some species.* However, it has a contrasting effect on the presence of some others.

This study showed that the increase in the abundance and richness of plant species has a positive effect on ants' abundance and richness. However, the gradients of soil elements' changes have a much more significant effect on ants' abundance and richness than vegetation change.

We found that, although a few species have a lesser presence in these regions, generally the ant species have adapted to the particular environmental conditions, such as existing conditions of the areas under this study, as well as *Cataglyphis bellicosus* having been collected from most sites of all four habitats.

Lastly, due to a lack of sufficient knowledge of ants in Iran, it is essential to highlight that the study's region has a high potential for further studies.

## Supplementary Material

2FF2F0E5-AFC6-5EA4-82FE-B527F5065217 10.3897/BDJ.9.e54843.suppl1 Supplementary material 1 The abundance of species in different habitats Data type Appendix Brief description The abundance of species in different habitats. File: oo_412567.pdf https://binary.pensoft.net/file/412567 Mohseni and Pashaei Rad

E43B2128-7B75-5E3F-967D-8FB74385063C 10.3897/BDJ.9.e54843.suppl2 Supplementary material 2 Variation in the rate of each environmental variable measured in soil of each site within each habitat type. Data type Table Brief description Values are estimated with maximum accuracy. File: oo_496736.docx https://binary.pensoft.net/file/496736 Mohseni and Pashaei Rad

79E9092C-C2AF-5F10-A8C9-0B5EE915BD55 10.3897/BDJ.9.e54843.suppl3 Supplementary material 3 Results of SIMPER analysis showing the contribution of top-ten ant species differentiating assemblage structures of habitats Data type Table File: oo_496735.docx https://binary.pensoft.net/file/496735 Mohseni and Pashaei Rad

## Figures and Tables

**Table 1.** Habitat characteristics of the study sites. Note: The temperature ranged between 30°C and 55°C (as the highest range) in dry months and 28°C (as the lowest range) to 35°C in rainy months amongst sites.

**Sites**	**Latitude (N) and Longitude (E)**	**Temperature Ranges** (With mean annual temperature/ MAT)	**Elevation**	**Humidity Ranges**	**Climate**	**Vegetation** (% estimation of vegetation cover)
Salt Lake (a)	34°58'03.4"N, 50°54'05.8"E	32-50°C MAT 42°C ± 2	804 m	7-12%	Dry and desert	poor (10%)
Historic caravanserai of Sadrabad (b)	34°53'05.3"N, 51°04'41.1"E	33-55°C MAT 40°C ± 2	805 m	6-9%	Dry and desert	Quite poor (5%)
Salty-lands of Qom Rood (c)	34°32'48.5"N, 50°14'27.0"E	29-46°C MAT 37°C ± 2	1705 m	15-17%	Hot and dry	Fairly poor (15%-20%)
Tagharood industrial area (d)	34°45'42.0"N, 50°30'53.9"E	35-39°C MAT 36°C ± 1	980 m	11-16%	Dry and semi-desert	Fairly poor (15%)
Cheshmeh Palang Village (e)	34°58'06.8"N, 50°47'12.2"E	33-37°C MAT 35°C ± 0.5	1008 m	8-10%	Dry and desert	Low-variety, Mostly sporadic grassland (25%)
Darbandshoor Mount (f)	34°25'50.9"N, 50°38'46.9"E	30-42°C MAT 35°C ± 2	1259 m	12-14%	Dry and desert	Low-variety, Mostly grassland (20%)
Shah Ismail shrine (g)	34°20'25.5"N, 50°59'51.4"E	32-36°C MAT 35°C ± 1	1662 m	15-16%	Hot and dry	Low-variety, Mostly grassland (20%)
Chalk mine (h)	34°14'24.7"N, 50°35'48.5"E	34-38°C MAT 35°C ± 1	1589 m	8-11%	Hot and dry	high-variety (30%)
Kebar Dam (i)	34°28'09.1"N, 51°00'46.0"E	37-43°C MAT 39°C ± 1	1017 m	15-20%	Semi-hot and semi-mild	Semi dense, Mostly grassland (30%-35%)
Ghahan Village (j)	34°43'43.0"N, 50°16'03.3"E	33-36°C MAT 35°C ± 0.5	1530 m	14-25%	Semi-hot and semi-mild	semi-variety and semi-dense (35%)
Cheshme Ali Village (k)	34°22'05.6"N, 50°34'57.2"E	34-37°C MAT 35°C ± 1	1216 m	13-15%	Hot and semi-dry	Highly-dense with semi-variety (45%)
Ghadir Forest Park (l)	34°35'00.3"N, 50°44'54.3"E	32-35°C MAT 34°C ± 0.5	938 m	14-19%	Hot and semi-dry	Highly-dense with semi-variety (40%)
Varzaneh Village (m)	34°33'41.1"N, 50°18'32.2"E	32-38°C MAT 35°C ± 1	1606 m	15-18%	Semi-hot and semi-mild	Highly-dense with rich and high-variety (60%)
Qanavat City (n)	34°36'31.5"N, 51°01'05.7"E	34-35°C MAT 35°C ± 0.5	880 m	13-17%	Semi-hot and semi-dry	Highly-dense with Low-variety (45%)
Dastjerd City (o)	34°43'33.3"N, 51°03'57.7"E	29-40°C MAT 34°C ± 2	844 m	14-27%	Hot and dry	Highly-dense with Low-variety (35%)
Qom City (p)	34°37'30.2"N, 50°53'17.7"E	30-37°C MAT 35°C ± 2	956 m	14-19%	Hot and semi-dry	Fairly poor but with high variety (40%)
Kahak City (q)	34°23'51.6"N, 50°51'54.8"E	32-36°C MAT 34°C ± 1	1780 m	16-30%	Semi-hot and semi-mild	Fairly poor but with high variety (45%)
Salafchegan City (r)	34°28'37.4"N, 50°27'55.8"E	36-37°C MAT 36°C ± 0.5	1375 m	16-25%	Hot and dry	Fairly poor but with low variety (30%)
Kamkar Castle (s)	34°39'52.8"N, 50°49'56.8"E	36-38°C MAT 36°C ± 0.5	928 m	14-20%	Hot and semi-dry	Fairly poor but with high variety (40%)
Jafariyeh City (t)	34°46'26.7"N, 50°29'40.9"E	28-34°C MAT 32°C ± 1	986 m	11-12%	Hot and dry	Poor but with high variety (20%)

**Table 2.** Types of vegetation available in the sampling areas (species are written in subfamily order). Frequencies of observed plant specimens in the sites are shown with (*).

**Nom.**	**Habitats**	**Types of vegetation**	**Plant species**
1	Desert	Poor vegetation cover, generally rough and harsh plants which include Cacti and Succulents, Wildflowers and Shrubs. The plants of this habitat are incredibly compatible with drought, scorching days and freezing nights.	*Alhagi camelorum* (28), *Alhagi maurorum* (8), *Prosopis farcta* (13), *Artemisia sieberi* (14), *Scariola orientalis* (12), *Launaea acanthodes* (12), *Arthrocnemum macrostachyum* (8), *Halocnemum strobilaceum* (18), *Halopeplis perfoliate (9)*, *Atriplex prostrata* (16), *Chenopodium album* (10), *Bassia indica* (13), *Haloxylon salicornicum* (21), *Anabasis setifera* (4), *Salsola stocksii* (9), *Seidlitzia Rosmarinus*(17), *Suaeda vermiculata* (12), *Aeluropus lagopoides* (6), *Cressa cretica* (14), *Chrozophora sabulosa* (14), *Chrozophora tinctorial* (10)
2	Mountain	Semi-high variety of vegetation cover, species include shrubs, perennial grasses, forbs, cushion plants, lichens and, in some cases, trees. Mountain plants are adapted to the harsh conditions of the mountainous environment, which include low temperatures, dryness, ultraviolet radiation, wind, drought, poor nutritional soil and a short growing season.	*Alhagi camelorum* (25), *Alhagi maurorum* (22), *Alhagi persarum* (16), *Astragalus verus* (12), *Prosopis farcta* (9), *Artemisia aucheri* (11), *Artemisia sieberi* (12), *Echinops ritro* (18), *Launaea acanthodes* (11), *Scariola orientalis* (8), *Arthrocnemum macrostachyum* (3), *Halopeplis perfoliate* (6), *Bassia indica* (6), *Atriplex prostrata* (9), *Chenopodium album* (12), *Salsola stocksii* (6), *Salsola* sp. (2), *Cressa cretica* (6), *Chrozophora tinctorial* (3), *Sporobolus spicatus* (15), *Zygophyllum simplex* (19), *Peganum harmala* (3), *Rheum ribes* (11), *Acanthophyllum microcephalum* (3), *Teucrium polium* (7), *Melica persica* (8), *Sameraria nummularia* (8), *Echinophora platyloba* (7), *Cupressus atlantica* (17), *Cupressus duclouxiana* (11), *Cupressus torulosa* (7)
3	Plain and Rural	Highly-dense with rich and high-variety of vegetation cover. Due to the favourable environmental conditions, almost all kinds of plant species are apparent.	*Alhagi persarum* (9), *Astragalus verus* (18), *Lycium edgeworthii* (12), *Carex divisa* (17), *Scirpoides holoschoenus* (27), *Juncus inflexus* (13), *Aeluropus lagopoides* (6), *Aeluropus littoralis* (22), *Melica persica* (38), *Stipa hohenackeriana* (17), *Halanthium purpureum* (24), *salsola imbricate* (31), *Artemisia sieberi* (22), *Andrachne fruticulosa* (8), *Pistacia atlantica* (9), *Acer monspessulanum* (14), *Prunus scoparia* (26), *Onosma microcarpum* (18), *Ajuga chamaecistus* (15), *Teucrium orientalis* (19), *Teucrium polium* (16), *Acanthophyllum microcephalum* (8), *Stachys acerosa* (15), *Andrachne fruticulosa* (9), *Sameraria nummularia* (8), *Rubia albicaulis* (13), *Actinostrobus arenarius* (17), *Callitris columellaris* (12), *Cupressus chengiana* (21), *Cupressus torulosa* (14), *Juglans regia* (14)
4	Urban	Fairly poor, but with high variety of vegetation cover. Due to the favourable environmental conditions, many kinds of plant species are apparent.	*Alhagi camelorum* (22), *Alhagi maurorum* (18), *Prosopis farcta* (16), *Launaea acanthodes* (11), *Scariola orientalis* (14), *Bassia indica* (25), *Atriplex prostrata* (9), *Chenopodium album* (10), *Salsola stocksii* (21), *Chrozophora tinctorial* (17), *Frankenia pulverulenta* (18), *Artemisia sieberi* (9), *Lactuca orientalis* (22), *Actinostrobus arenarius* (21), *Callitris preissii* (16), *Callitris rhomboidei* (19), *Cupressus duclouxiana* (27)
