# Supplementary material for: The effect of edaphic factors on the distribution and abundance of ants (Hymenoptera: Formicidae) in Iran
Source: Biodivers Data J. 2021 Jan 15;9:e54843. doi: 10.3897/BDJ.9.e54843 (PMC7822805; doi:10.3897/BDJ.9.e54843)
Supplement: Supplementary material 1 — The abundance of species in different habitats [file bdj-09-e54843-s001.pdf]

The abundance of species in different habitats

	Desert habitat						Mountainous and submontane habitat						Plain and Rural habitat						Urban habitat					
**Species**	Salt Lake	Historic Caravanserai of Sadrabad	Saltylands of Qom Rood	Tagharood Industrial Area	Cheshmeh Palang village	SUM	Darbandshoor Mount	Shah Ismaeil Shrine	Chalk mine	Kebar Dam	Ghahan village	SUM	Cheshme Ali village	Ghadir forest Park	Varzaneh village	Qanavat city	Dastjerd city	SUM	Qom city	Kahak city	Salafchegan city	Kamkar castle	Jafariyeh city	SUM
***Messor mediorubra* (Forel, 1905)**	-	-	2	-	-	2	-	2	-	-	3	5	-	1	3	3	2	9	-	4	-	2	2	8
***Messor ebeninus* (Santschi, 1927)**	-	-	-	2	-	2	1	1	-	-	-	2	-	2	-	-	2	4	-	2	-	-	3	5
***Messor minor* (André, 1883)**	-	-	-	-	-	-	-	-	-	-	-	-	-	-	-	-	-	-	-	1	-	-	3	4
***Messor galla* (Mayr, 1904)**	-	-	-	-	-	-	-	-	-	-	-	-	-	-	-	-	-	-	-	-	-	-	1	1
***Messor rufotestaceus* (Foerster, 1850)**	-	-	-	-	-	-	-	-	-	-	-	-	-	-	-	-	-	-	4	-	-	-	-	4
***Messor* sp. (Forel, 1890)**	-	-	-	-	-	-	-	-	-	-	-	-	1	-	-	-	-	1	-	-	-	-	-	-
***Monomorium indicum* (Forel, 1902)**	1	-	-	3	2	6	4	-	3	-	-	7	-	2	-	2	-	4	-	-	-	2	1	3
***Monomorium pharaonic* (Linnaeus, 1758)**	-	-	-	-	-	-	-	-	-	-	-	-	-	2	-	-	-	2	-	-	-	-	-	-
***Monomorium kusnezowi* (Santschi, 1928)**	1	-	3	-	-	4	-	-	-	-	-	-	-	1	-	-	-	1	-	3	-	3	-	6
***Monomorium subopacum* (Smith, F., 1858)**	-	-	1	-	-	1	-	-	-	-	-	-	-	-	-	-	-	-	-	-	2	-	-	2
***Pheidole teneriffana* (Forel, 1893)**	-	-	-	-	-	-	-	-	-	-	-	-	-	-	-	-	-	-	9	-	-	-	-	9
***Pheidole megacephala* (Fabricius, 1793)**	-	-	-	-	1	1	-	-	-	-	-	-	-	2	-	-	-	2	-	-	-	-	-	-
***Pheidole pallidula* (Nylander, 1849)**	-	-	-	-	-	-	-	-	-	-	-	-	-	2	3	-	-	5	5	3	-	-	-	8
***Tetramorium moravicum* (Kratochvíl, 1941)**	-	-	-	-	1	1	-	-	-	-	-	-	-	-	-	-	-	-	-	-	-	-	-	-
***Tetramorium* sp. (Mayr, 1855)**	-	-	-	-	2	2	2	1	-	-	-	3	-	-	-	-	-	-	-	3	-	-	-	3
***Crematogaster oasium* (Santschi, 1911)**	-	-	-	-	-	-	-	-	-	-	-	-	-	-	4	-	-	4	-	-	-	-	-	-

***Cardiocondyla ulianini* (Emery, 1889)**	-	-	-	-	-	-	-	-	1	-	-	1	-	-	2	-	-	2	-	2	-	-	-	2
***Cardiocondyla stambuloffi* (Forel, 1892)**	-	-	-	-	-	-	-	-	-	-	-	-	-	-	-	-	-	-	-	1	-	-	-	1
***Cataglyphis niger*(André, 1881)**	-	-	-	-	-	-	3	-	-	-	-	3	-	-	-	2	-	2	-	1	-	-	-	1
***Cataglyphis bellicosus* (Karavaiev, 1924)**	1	-	-	1	-	2	2	3	2	5	2	14	2	2	4	-	3	11	-	3	3	-	4	10
***Cataglyphis setipes* (Forel, 1894)**	-	-	3	3	-	6	-	3	-	-	4	7	-	-	4	3	3	10	-	4	4	-	3	11
***Cataglyphis altisquamis* (André, 1881)**	-	-	-	-	-	-	-	-	-	-	-	-	-	-	-	-	1	1	-	-	-	-	-	-
***Cataglyphis lividus* (André, 1881)**	3	1	-	3	2	9	-	2	4	-	-	6	3	-	3	-	4	10	-	-	4	-	-	4
***Cataglyphis frigidus* (André, 1881)**	-	-	-	-	-	-	-	-	1	-	-	1	-	-	-	-	-	-	-	-	-	-	-	-
***Lepisiota dolabellae* (Forel, 1911)**	-	-	4	-	-	4	-	2	3	3	4	12	2	-	-	-	3	5	4	3	-	3	4	14
***Lepisiota bipartite* (Smith, F., 1861)**	-	-	-	-	-	-	-	-	-	1	-	1	-	-	-	-	-	-	-	-	-	-	-	-
***Paratrechina longicornis* (Latreille, 1802)**	-	-	-	-	-	-	-	-	-	-	-	-	-	-	-	-	-	-	3	-	-	-	-	3
***Plagiolepis abyssinica* (Forel, 1894)**	-	-	-	-	-	-	-	-	-	-	-	-	-	-	3	-	-	3	-	-	-	-	-	-
***Camponotus flavomarginatus* (Mayr, 1862)**	-	-	-	-	-	-	-	-	-	-	-	-	-	-	-	-	1	1	-	-	-	-	-	-
***Camponotus kurdistanicus* (Emery, 1898)**	-	-	1	-	-	1	-	-	-	-	-	-	-	-	-	-	1	1	-	-	-	-	-	-
***Lasius alienus* (Foerster, 1850)**	-	-	-	-	-	-	-	-	-	-	-	-	-	-	2	-	-	2	5	-	-	-	-	5
***Tapinoma simrothi* (Krausse, 1911)**	-	-	-	-	-	-	-	3	-	-	-	3	2	4	3	-	-	9	4	3	-	2	3	12
**SUM**	6	1	14	12	8	41	12	17	14	9	13	65	10	18	31	10	20	89	34	33	13	12	24	116
