# Supplementary material for: The effect of edaphic factors on the distribution and abundance of ants (Hymenoptera: Formicidae) in Iran
Source: Biodivers Data J. 2021 Jan 15;9:e54843. doi: 10.3897/BDJ.9.e54843 (PMC7822805; doi:10.3897/BDJ.9.e54843)
Supplement: Supplementary material 2 — Variation in the rate of each environmental variable measured in soil of each site within each habitat type. [file bdj-09-e54843-s002.docx]

Variation in the rate of each environmental variable measured in soil of each station within each habitat type. (values are estimated with maximum accuracy.)

| **Habitat type** | **Station** | **SAR** | **Na**  **mg/lit** | **Ca**  **mg/lit** | **Mg**  **mg/lit** | **K (ava)**  **ppm** | **P (ava)**  **ppm** | **OC**  **%** | **Total N %** | **pH** | **Salinity (EC)**  **ds/m** | **Sand %** | **Silt %** | **Clay %** | **Soil texture** |
| --- | --- | --- | --- | --- | --- | --- | --- | --- | --- | --- | --- | --- | --- | --- | --- |
| Desert habitat | Salt Lake | 6.26 | 46.00 | 72.00 | 36.00 | 244.20 | 5.80 | 3.05 | 0.26 | 7.11 | 153.15 | 0.00 | 0.00 | 0.00 | Flocculated |
|  | Historic Caravanserai of Sadr Abad | 9.51 | 43.00 | 18.00 | 23.00 | 508.40 | 15.40 | 0.69 | 0.07 | 7.86 | 83.58 | 58.00 | 26.00 | 16.00 | Sandy loam |
|  | Saltylands of Qom Rood | 4.60 | 18.00 | 19.00 | 18.00 | 713.20 | 9.30 | 0.25 | 0.03 | 7.26 | 10.05 | 65.00 | 30.00 | 17.00 | Loam |
|  | Tagharood Industrial Area | 3.50 | 12.00 | 11.00 | 7.00 | 541.20 | 14.50 | 0.68 | 0.06 | 6.10 | 16.75 | 70.00 | 30.00 | 11.00 | Silty clay |
|  | Cheshmeh Palang Village | 3.60 | 13.00 | 9.00 | 11.00 | 679.20 | 10.30 | 0.29 | 0.03 | 6.83 | 38.41 | 68.00 | 15.00 | 22.00 | Sandy loam |
| Minimum |  | 3.50 | 12.00 | 9.00 | 7.00 | 244.20 | 5.80 | 0.25 | 0.03 | 6.10 | 10.05 | 0.00 | 0.00 | 0.00 |  |
| Maximum |  | 9.51 | 46.00 | 72.00 | 36.00 | 713.20 | 15.40 | 3.05 | 0.26 | 7.86 | 153.15 | 70.00 | 30.00 | 22.00 |  |
| Mean |  | 5.49 | 26.40 | 25.80 | 19.00 | 537.24 | 11.06 | 0.99 | 0.09 | 7.03 | 60.39 | 52.20 | 20.20 | 13.20 |  |
| SD |  | 2.50 | 16.71 | 26.19 | 11.34 | 185.63 | 3.94 | 1.17 | 0.10 | 0.64 | 59.30 | 29.53 | 12.85 | 8.35 |  |
| Mountainous and submontane habitat | Darbandshoor Mount | 2.93 | 8.00 | 9.00 | 6.00 | 274.60 | 7.20 | 0.18 | 0.02 | 12.04 | 22.14 | 74.00 | 16.00 | 10.00 | Sandy loam |
|  | Shah Ismail's Shrine | 4.90 | 25.00 | 21.00 | 10.00 | 598.30 | 5.80 | 0.24 | 0.02 | 7.13 | 37.36 | 75.00 | 25.00 | 10.00 | Silty loam |
|  | Chalk Mine | 8.43 | 40.00 | 32.00 | 13.00 | 132.60 | 2.40 | 0.18 | 0.02 | 7.58 | 8.48 | 82.00 | 8.00 | 10.00 | Loamy sand |
|  | Kebar Dam | 5.20 | 34.00 | 17.00 | 21.00 | 880.10 | 17.33 | 0.53 | 0.04 | 7.03 | 46.30 | 70.00 | 52.00 | 14.00 | Sandy loam |
|  | Ghahan Village | 4.70 | 20.00 | 22.00 | 29.00 | 1129.60 | 13.50 | 0.64 | 0.07 | 8.12 | 33.70 | 70.00 | 36.00 | 18.00 | Loam |
| Minimum |  | 2.93 | 8.00 | 9.00 | 6.00 | 132.60 | 2.40 | 0.18 | 0.02 | 7.03 | 8.48 | 70.00 | 8.00 | 10.00 |  |
| Maximum |  | 8.43 | 40.00 | 32.00 | 29.00 | 1129.60 | 17.33 | 0.64 | 0.07 | 12.04 | 46.30 | 82.00 | 52.00 | 18.00 |  |
| Mean |  | 5.23 | 25.40 | 20.20 | 15.80 | 603.04 | 9.25 | 0.35 | 0.03 | 8.38 | 29.60 | 74.20 | 27.40 | 12.40 |  |
| SD |  | 2.00 | 12.44 | 8.35 | 9.20 | 413.29 | 6.05 | 0.22 | 0.02 | 2.09 | 14.64 | 4.92 | 17.26 | 3.58 |  |
| Plain and rural habitat | Cheshme Ali Village | 3.81 | 18.00 | 20.00 | 19.00 | 811.30 | 8.32 | 0.22 | 0.02 | 7.67 | 45.68 | 55.00 | 20.00 | 15.00 | Clay Loam |
|  | Ghadir Forest Park | 6.12 | 38.00 | 46.00 | 23.00 | 572.30 | 11.20 | 0.52 | 0.06 | 6.90 | 88.36 | 48.00 | 50.00 | 16.00 | Silty loam |
|  | Varzaneh Village | 3.45 | 16.00 | 25.00 | 18.00 | 1448.50 | 17.80 | 0.69 | 0.07 | 7.34 | 5.58 | 44.00 | 42.00 | 14.00 | Loam |
|  | Qanavat City | 3.20 | 12.00 | 18.00 | 10.00 | 864.00 | 33.00 | 0.69 | 0.07 | 7.81 | 39.63 | 60.00 | 28.00 | 12.00 | Sandy loam |
|  | Dastjerd City | 3.53 | 12.00 | 14.00 | 9.00 | 305.20 | 11.80 | 0.27 | 0.03 | 7.56 | 34.26 | 26.00 | 52.00 | 22.00 | Silty loam |
| Minimum |  | 3.20 | 12.00 | 14.00 | 9.00 | 305.20 | 8.32 | 0.22 | 0.02 | 6.90 | 5.58 | 26.00 | 20.00 | 12.00 |  |
| Maximum |  | 6.12 | 38.00 | 46.00 | 23.00 | 1448.50 | 33.00 | 0.69 | 0.07 | 7.81 | 88.36 | 60.00 | 52.00 | 22.00 |  |
| Mean |  | 4.02 | 19.20 | 24.60 | 15.80 | 800.26 | 16.42 | 0.48 | 0.05 | 7.46 | 42.70 | 46.60 | 38.40 | 15.80 |  |
| SD |  | 1.19 | 10.83 | 12.60 | 6.06 | 424.69 | 9.89 | 0.22 | 0.02 | 0.36 | 29.80 | 13.07 | 13.96 | 3.77 |  |
| Urban habitat | Qom City | 5.31 | 34.00 | 51.00 | 31.00 | 630.40 | 7.80 | 0.60 | 0.06 | 7.42 | 115.31 | 40.00 | 40.00 | 20.00 | Loam |
|  | Kahak City | 4.90 | 26.00 | 38.00 | 27.00 | 978.70 | 12.60 | 0.50 | 0.05 | 7.30 | 65.89 | 45.00 | 45.00 | 18.00 | Loam |
|  | Salafchegan City | 3.62 | 14.00 | 28.00 | 22.00 | 624.50 | 8.90 | 0.31 | 0.03 | 7.65 | 22.35 | 52.00 | 20.00 | 16.00 | Silty loam |
|  | Kamkar Castle | 5.30 | 35.00 | 48.00 | 30.00 | 702.10 | 15.80 | 0.61 | 0.06 | 7.40 | 120.33 | 30.00 | 52.00 | 20.00 | Silty clay loam |
|  | Jafariyeh City | 3.36 | 15.00 | 18.00 | 26.00 | 718.40 | 6.90 | 0.17 | 0.02 | 7.18 | 66.17 | 65.00 | 16.00 | 20.00 | Sandy loam |
| Minimum |  | 3.36 | 14.00 | 18.00 | 22.00 | 624.50 | 6.90 | 0.17 | 0.02 | 7.18 | 22.35 | 30.00 | 16.00 | 16.00 |  |
| Maximum |  | 5.31 | 35.00 | 51.00 | 31.00 | 978.70 | 15.80 | 0.61 | 0.06 | 7.65 | 120.33 | 65.00 | 52.00 | 20.00 |  |
| Mean |  | 4.50 | 24.80 | 36.60 | 27.20 | 730.82 | 10.40 | 0.44 | 0.05 | 7.39 | 78.01 | 46.40 | 34.60 | 18.80 |  |
| SD |  | 0.94 | 10.03 | 13.78 | 3.56 | 144.75 | 3.72 | 0.19 | 0.02 | 0.17 | 40.52 | 13.13 | 15.81 | 1.79 |  |
