# Supplementary material for: The effect of edaphic factors on the distribution and abundance of ants (Hymenoptera: Formicidae) in Iran
Source: Biodivers Data J. 2021 Jan 15;9:e54843. doi: 10.3897/BDJ.9.e54843 (PMC7822805; doi:10.3897/BDJ.9.e54843)
Supplement: Supplementary material 3 — Results of SIMPER analysis showing the contribution of top-ten ant species differentiating assemblage structures of habitats [file bdj-09-e54843-s003.docx]

Results of SIMPER analysis showing the contribution of top-ten ant species differentiating assemblage structures of habitats.

| Comparisons | Ant species | Average abundance in DH | Average abundance in MSH | Average dissimilarity | Dissimilarity /SD | Contribution% | Cumulative contribution% |
| --- | --- | --- | --- | --- | --- | --- | --- |
| Desert habitat (DH)  vs  mountainous and submontane habitat (MSH)  Average dissimilarity = 73.57% | *Cataglyphis bellicosus* | 0.4 | 1.64 | 10.34 | 1.35 | 14.05 | 14.05 |
|  | *Lepisiota dolabellae* | 0.4 | 1.38 | 10.09 | 1.26 | 13.71 | 27.76 |
|  | *Cataglyphis lividus* | 1.18 | 0.68 | 8.01 | 1.35 | 10.89 | 38.66 |
|  | *Monomorium indicum* | 0.83 | 0.75 | 7.01 | 1.11 | 9.52 | 48.18 |
|  | *Cataglyphis setipes* | 0.69 | 0.75 | 6.51 | 0.9 | 8.85 | 57.03 |
|  | *Messor mediorubra* | 0.28 | 0.63 | 4.99 | 0.81 | 6.79 | 63.81 |
|  | *Tetramorium* sp*.* | 0.28 | 0.48 | 4.21 | 0.81 | 5.72 | 69.53 |
|  | *Monomorium kusnezowi* | 0.55 | 0 | 3.7 | 0.78 | 5.03 | 74.56 |
|  | *Messor ebeninus* | 0.28 | 0.4 | 3.69 | 0.89 | 5.02 | 79.58 |
|  | *Cataglyphis niger* | 0 | 0.35 | 2.75 | 0.47 | 3.74 | 83.32 |
|  | Ant species | Average abundance in DH | Average abundance in PRH | Average dissimilarity | Dissimilarity /SD | Contribution% | Cumulative contribution% |
| Desert habitat (DH)  vs  plain and rural habitat (PRH)  Average dissimilarity = 73.18% | *Messor mediorubra* | 0.28 | 1.18 | 6.68 | 1.17 | 9.13 | 9.13 |
|  | *Cataglyphis bellicosus* | 0.4 | 1.31 | 6.45 | 1.49 | 8.82 | 17.95 |
|  | *Cataglyphis setipes* | 0.69 | 1.09 | 6.17 | 0.93 | 8.43 | 26.38 |
|  | *Tapinoma simrothi* | 0 | 1.03 | 6.14 | 1.1 | 8.39 | 34.77 |
|  | *Cataglyphis lividus* | 1.18 | 1.09 | 5.73 | 1.15 | 7.83 | 42.6 |
|  | *Monomorium indicum* | 0.83 | 0.57 | 5.03 | 1.01 | 6.87 | 49.47 |
|  | *Lepisiota dolabellae* | 0.4 | 0.63 | 4.99 | 0.88 | 6.82 | 56.29 |
|  | *Messor ebeninus* | 0.28 | 0.57 | 3.61 | 0.84 | 4.93 | 61.22 |
|  | *Monomorium kusnezowi* | 0.55 | 0.2 | 3.43 | 0.85 | 4.69 | 65.91 |
|  | *Pheidole pallidula* | 0 | 0.63 | 3.11 | 0.78 | 4.25 | 70.16 |
|  | Ant species | Average abundance in MSH | Average abundance in PRH | Average dissimilarity | Dissimilarity /SD | Contribution% | Cumulative contribution% |
| Mountainous and submontane habitat (MSH)  vs  plain and rural habitat (PRH)  Average dissimilarity = 63.15% | *Lepisiota dolabellae* | 1.38 | 0.63 | 5.63 | 1.17 | 8.91 | 8.91 |
|  | *Cataglyphis lividus* | 0.68 | 1.09 | 5.42 | 1.07 | 8.58 | 17.49 |
|  | *Cataglyphis setipes* | 0.75 | 1.09 | 5.4 | 1.02 | 8.55 | 26.04 |
|  | *Tapinoma simrothi* | 0.35 | 1.03 | 5.18 | 1.12 | 8.2 | 34.24 |
|  | *Messor mediorubra* | 0.63 | 1.18 | 5.08 | 1.12 | 8.04 | 42.28 |
|  | *Monomorium indicum* | 0.75 | 0.57 | 4.79 | 1.03 | 7.59 | 49.87 |
|  | *Cataglyphis bellicosus* | 1.64 | 1.31 | 3.62 | 0.74 | 5.74 | 55.61 |
|  | *Messor ebeninus* | 0.4 | 0.57 | 3.34 | 1.06 | 5.28 | 60.89 |
|  | *Cataglyphis niger* | 0.35 | 0.28 | 3.14 | 0.68 | 4.98 | 65.87 |
|  | *Pheidole pallidula* | 0 | 0.63 | 2.76 | 0.79 | 4.38 | 70.24 |
|  | Ant species | Average abundance in DH | Average abundance in UH | Average dissimilarity | Dissimilarity /SD | Contribution% | Cumulative contribution% |
| Desert habitat (DH)  vs  urban habitat (UH)  Average dissimilarity = 77.74% | *Lepisiota dolabellae* | 0.4 | 1.49 | 7.33 | 1.3 | 9.43 | 9.43 |
|  | *Tapinoma simrothi* | 0 | 1.38 | 7.29 | 1.7 | 9.38 | 18.81 |
|  | *Cataglyphis lividus* | 1.18 | 0.4 | 6.51 | 1.56 | 8.37 | 27.18 |
|  | *Cataglyphis setipes* | 0.69 | 1.15 | 6.08 | 0.93 | 7.82 | 35 |
|  | *Cataglyphis bellicosus* | 0.4 | 1.09 | 5.9 | 1.15 | 7.59 | 42.59 |
|  | *Messor mediorubra* | 0.28 | 0.97 | 5.03 | 1.05 | 6.47 | 49.06 |
|  | *Monomorium kusnezowi* | 0.55 | 0.69 | 4.7 | 0.89 | 6.05 | 55.11 |
|  | *Monomorium indicum* | 0.83 | 0.48 | 4.47 | 1.02 | 5.75 | 60.86 |
|  | *Pheidole pallidula* | 0 | 0.79 | 3.53 | 0.75 | 4.55 | 65.41 |
|  | *Messor ebeninus* | 0.28 | 0.63 | 3.41 | 0.84 | 4.39 | 69.79 |
|  | Ant species | Average abundance in MSH | Average abundance in UH | Average dissimilarity | Dissimilarity /SD | Contribution% | Cumulative contribution% |
| Mountainous and submontane habitat (MSH)  vs  urban habitat (UH)  Average dissimilarity = 64.99% | *Tapinoma simrothi* | 0.35 | 1.38 | 5.77 | 1.45 | 8.87 | 8.87 |
|  | *Cataglyphis setipes* | 0.75 | 1.15 | 5.22 | 0.98 | 8.03 | 16.9 |
|  | *Cataglyphis bellicosus* | 1.64 | 1.09 | 4.64 | 1.08 | 7.15 | 24.05 |
|  | *Messor mediorubra* | 0.63 | 0.97 | 4.4 | 1.08 | 6.77 | 30.82 |
|  | *Monomorium indicum* | 0.75 | 0.48 | 4.35 | 1.03 | 6.7 | 37.52 |
|  | *Cataglyphis lividus* | 0.68 | 0.4 | 4.23 | 0.79 | 6.51 | 44.03 |
|  | *Lepisiota dolabellae* | 1.38 | 1.49 | 3.88 | 0.81 | 5.98 | 50 |
|  | *Monomorium kusnezowi* | 0 | 0.69 | 3.48 | 0.74 | 5.36 | 55.36 |
|  | *Messor ebeninus* | 0.4 | 0.63 | 3.25 | 1.07 | 5 | 60.36 |
|  | *Pheidole pallidula* | 0 | 0.79 | 3.18 | 0.76 | 4.89 | 65.25 |
|  | Ant species | Average abundance in PRH | Average abundance in UH | Average dissimilarity | Dissimilarity /SD | Contribution% | Cumulative contribution% |
| Plain and rural habitat (PRH)  vs  urban habitat (UH)  Average dissimilarity = 63.89% | *Lepisiota dolabellae* | 0.63 | 1.49 | 4.78 | 1.24 | 7.49 | 7.49 |
|  | *Cataglyphis lividus* | 1.09 | 0.4 | 4.58 | 1.03 | 7.16 | 14.65 |
|  | *Cataglyphis setipes* | 1.09 | 1.15 | 4.29 | 0.96 | 6.72 | 21.37 |
|  | *Cataglyphis bellicosus* | 1.31 | 1.09 | 3.98 | 1.04 | 6.23 | 27.6 |
|  | *Tapinoma simrothi* | 1.03 | 1.38 | 3.96 | 1.04 | 6.2 | 33.8 |
|  | *Messor mediorubra* | 1.18 | 0.97 | 3.75 | 1.08 | 5.87 | 39.68 |
|  | *Pheidole pallidula* | 0.63 | 0.79 | 3.63 | 0.99 | 5.69 | 45.36 |
|  | *Monomorium kusnezowi* | 0.2 | 0.69 | 3.22 | 0.84 | 5.03 | 50.4 |
|  | *Messor ebeninus* | 0.57 | 0.63 | 3.07 | 0.95 | 4.8 | 55.2 |
|  | *Monomorium indicum* | 0.57 | 0.48 | 3.05 | 0.91 | 4.77 | 59.97 |
